# Identifying Features of Cardiac Disease Phenotypes Based on Mechanical Function in a Catecholaminergic Polymorphic Ventricular Tachycardia Model

**DOI:** 10.3389/fbioe.2022.873531

**Published:** 2022-05-10

**Authors:** A. Stempien, M. Josvai, W. J. de Lange, J. J. Hernandez, J. Notbohm, T. J. Kamp, H. H. Valdivia, L. L. Eckhardt, K. R. Maginot, J. C. Ralphe, W. C. Crone

**Affiliations:** ^1^ Department of Biomedical Engineering, University of Wisconsin-Madison, Madison, WI, United States; ^2^ Wisconsin Institute for Discovery, University of Wisconsin-Madison, Madison, WI, United States; ^3^ Department of Pediatrics, University of Wisconsin School of Medicine and Public Health, Madison, WI, United States; ^4^ Department of Engineering Physics, University of Wisconsin-Madison, Madison, WI, United States; ^5^ Department of Medicine, Division of Cardiovascular Medicine, University of Wisconsin-Madison, Madison, WI, United States; ^6^ Department of Cell and Regenerative Biology, University of Wisconsin-Madison, Madison, WI, United States; ^7^ Cellular and Molecular Arrhythmia Research Program, Department of Medicine, University of Wisconsin-Madison, Madison, WI, United States

**Keywords:** iPSC-cardiomyocytes, disease model, contraction rate, DIC, mechanical strain, microcontact printing, 2D cell culture

## Abstract

Catecholaminergic polymorphic ventricular tachycardia (CPVT) is characterized by an arrhythmogenic mechanism involving disruption of calcium handling. This genetic disease can lead to sudden death in children and young adults during physical or emotional stress. Prior CPVT studies have focused on calcium handling, but mechanical functionality has rarely been investigated *in vitro*. In this research we combine stem cell-derived cardiomyocytes from a CPVT patient (RyR2-H2464D mutation) and a healthy familial control with an engineered culture platform to evaluate mechanical function of cardiomyocytes. Substrates with Young’s modulus ranging from 10 to 50 kPa were used in conjunction with microcontact printing of ECM proteins into defined patterns for subsequent attachment. Digital Image Correlation (DIC) was used to evaluate collections of contracting cells. The amplitude of contractile strain was utilized as a quantitative indicator of functionality and disease severity. We found statistically significant differences: the maximum contractile strain was consistently higher in patient samples compared to control samples on all substrate stiffnesses. Additionally, the patient cell line had a statistically significantly slower intrinsic contraction rate than the control, which agrees with prior literature. Differences in mechanical strain have not been previously reported, and hypercontractility is not a known characteristic of CPVT. However, functional changes can occur as the disease progresses, thus this observation may not represent behavior observed in adolescent and adult patients. These results add to the limited studies of mechanical function of CPVT CMs reported in literature and identify functional differences that should be further explored.

## Introduction

Catecholaminergic polymorphic ventricular tachycardia (CPVT) is an inherited disorder characterized by adrenergic stress-related tachycardia and possible sudden cardiac death (SCD), despite a structurally and functionally normal heart at rest (Pölönen et al., 2020). Two genetic pathologies have been identified for CPVT, the autosomal dominant form associated with mutations of the genes encoding ryanodine receptor 2 (RYR2) and involving mutations encoding cardiac calsequestrin (CASQ2) (Lahat et al., 2001; Priori et al., 2001). Despite CASQ2 variants generally causing an autosomal recessive form of CPVT arrhythmic phenotypes among heterozygotes have been reported (Ng et al., 2020). Mutations in the RyR2 gene are most common, having been found in approximately 55% of people with CPVT (Pérez-Riera et al., 2017). RyR2 is responsible for Ca^2+^ regulation in the sarcoplasmic reticulum; maintenance of the proper amount of Ca^2+^ is necessary to regulate contraction event timing (Medeiros-Domingo et al., 2009). The RYR2 channel has also been implicated in the intrinsic heart rate and rhythmicity in animal models, with loss-of-function mutations often resulting in lethal arrhythmias or SCD (Bround et al., 2012). More than 100 RyR2 and 12 CASQ2 mutations have been reported as causative to CPVT (Ackerman et al., 2011).

Complete knockout of the RyR2 receptor is reported to be embryonically lethal in mice, as the SR lacks the ability to release Ca^2+^ (Takeshima et al., 1998). Studies of transgenic mice with R4496C and R2474S RYR2 mutations found non-significant differences in cardiac mechanical function at baseline conditions, in comparison to a wild type control (Kushnir et al., 2010). Treatment with the nonselective beta-agonist isoproterenol or increased extracellular [Ca^2+^] resulted in a significant decrease in peak cardiac tension (mN/mm^2^) achieved in both atrial and ventricular trabeculae of transgenic mice (Ferrantini et al., 2016). Isoproterenol treatment has also been demonstrated to result in delayed afterdepolarizations (DADs) and arrhythmias in conditional RyR2 KO mice resulting in a 50% decrease in RyR2 expression, significantly decreasing the beat rate and cardiac output (ml/min) in comparison to wild type controls (Bround et al., 2012).

*In vitro* studies using hiPSC-CMs found similar results. Multiple studies using 2D culture systems reported significant differences in the intrinsic beat rate of WT hiPSC-CMs and hiPSC-CMs derived from donors with both the R420Q and V4563F RyR2 mutations (Novak et al., 2015; Ahola et al., 2018). Isoproterenol was also shown to significantly decrease the beat rate of *in vitro* iPSC-CMs carrying RYR2 mutations due to DADs resulting in arrhythmogenesis (Fatima et al., 2011; Novak et al., 2012). However, other aspects of mechanical function of cardiomyocytes beyond beat rate have not been well characterized. In this research we build on prior electrophysiological studies to investigate mechanical function using an iPSC line from a 19-year-old male patient with a CPVT mutation, and a healthy control line from the mother of the patient (Hernández et al., 2013; Hernandez, 2016).

Numerous *in vitro* systems have been developed to study cell-cell and cell-extracellular matrix (ECM) interactions (Schroer et al., 2019). The 2D culture system used in this study allows for a range of cues to be presented to the cells of interest and for imaging techniques that can quantify structural, mechanical, and electrical function. This experimental platform uses microcontact printing of ECM proteins in defined regions onto soft polymeric substrates. Lanes of narrow widths have been previously shown to promote elongation of CMs as well as alignment of sarcomeres in the cells (Salick et al., 2014). Using the same narrow lane width, a 15° chevron pattern was created that retains the dimensions of the lanes and thus the shape of the CMs while providing increased connectivity, which promotes synchronization of contraction across the substrate (Notbohm et al., 2019). This microenvironment produces aligned, electrically-coupled iPSC-CMs that exhibit anisotropic electrical impulse propagation within a platform suitable for microscopy techniques, allowing for mechanical analyses (Napiwocki et al., 2021a; Napiwocki et al., 2021b).

In addition to structural analysis of CMs, quantitative analysis of mechanical function can provide useful insight into contractile behavior of cardiomyocytes. Digital Image Correlation (DIC) can be utilized on seeded cells that have high contrast, thus no additional substrate fabrication is needed and a standard bright field microscope can be used. Video of spontaneous contraction events occurring within the cardiomyocyte syncytium are recorded, and the displacement field for the entire pattern can be measured. Principal strains are calculated from the displacements which ultimately capture strain along the length of and perpendicular to the major axis of the CM due to their aligned myofibrils, which provides quantification of the contraction mechanics on substrates of physiologically relevant stiffness. This analysis of CPVT hiPSC-CMs adds to the limited studies of mechanical function reported in literature and demonstrates the utility of this approach in quantifying mechanical function to understand phenotypic differences in cardiac disease.

## Methods

### Cell Culture

Human induced pluripotent stem cells (hiPSCs) were derived from a 19 year-old male patient diagnosed with CPVT and the patient’s mother. DNA testing confirmed the presence of the H2464D mutation in RyR2 in the patient. The patient’s mother, a 44-year-old female, was found to have normal phenotype and genotype (Hernández et al., 2013; Hernandez, 2016). RyR2-H2464D is a *de novo* missense mutation involving a single nucleotide replacement, C > G at position 7390 of the *RYR2* sequence (Hernández et al., 2013; Hernandez, 2016). Skin biopsies were taken to obtain skin fibroblasts, and three clones of hiPSCs per individual were obtained from Cellular Dynamics International (CDI; Madison, WI, United States), where episomal reprogramming was used to generate the hiPSCs (Yu et al., 2009). One clone of each line was used for experiments: 016 a.15 control line and 015 a.4 patient line.

One vial per cell line containing 1 × 10^6^ hiPSCs was thawed in mTESR media (STEMCELL Technologies Inc.) and centrifuged for 3 min at 300 rpm. The pellet of cells was resuspended in mTESR supplemented with 5 μM Y27632/ROCK inhibitor (Biogems). The cells were plated in a 6-well plate coated with Matrigel (Corning). Cells were passaged at 70%–80% confluence using the dissociation reagent Versene (Thermo Fisher Scientific). Versene was added for 4 min at room temperature before removing and re-seeding cells in mTeSR and ROCK. hiPSCs were passaged at least three times after thawing before beginning differentiation.

CMs were differentiated from pluripotent stem cells using a modified version of the small molecule Wnt-agonist method (Lian et al., 2012). Briefly, hiPSCs were passaged onto Matrigel coated 6-well plates and fed with mTesR medium until 100% confluent. On day 0 (3 days after passage) the medium was changed to RPMI (Thermo Fisher Scientific) supplemented with 2% B27 minus insulin (Thermo Fisher Scientific) and 12 μM of the GSK3 inhibitor CHIR 99021 (Selleck Chemicals). On day 1 at ± 2 h from the time CHIR was added, the cells were fed with RMPI with 2% B27 minus insulin. On day 3, 72 h from the time CHIR was added, cells were treated with RPMI supplemented with 2% B27-insulin and 5 μM of Wnt inhibitor IWP-4 (StemGent). On day 5, cells were fed with RPMI with 2% B27-insulin, then treated with RPMI containing 2% B27 complete on days 7, 9, 11, 13, and 15. Contractions began on day 10–13. Beginning on day 17 of culture, cells were purified using lactate medium consisting of RPMI without glucose (Life Technologies), 2% B-27 complete (Life Technologies) and 5 mM lactate (Sigma) (Tohyama et al., 2013). Cells were fed every other day for 10 days, and then changed to EB2 medium: DMEM/F12 (Invitrogen), 2% FBS (Invitrogen), 1% NEAA (Invitrogen), 0.5% GlutaMax (Lifetech) and 0.007% 2-Mercaptoethanol (Sigma), which was replaced every 3 days thereafter.

To seed on the patterned substrate, cells were rinsed with 1 ml PBS then exposed to TrypLE (Life Technologies) for 10–12 min to dissociate from the culture dish. The cell suspension was centrifuged for 5 min at 300 rpm to collect the CMs, and the resuspended in EB20 medium: DMEM/F12 (Invitrogen), 20% FBS (Invitrogen), 1% NEAA (Invitrogen), 0.5% GlutaMax (Lifetech) and 0.007% 2-Mercaptoethanol (Sigma). Samples are seeded onto the patterns at a density of 3,370 CMs/mm^2^ (120,000 CMs/35.6 mm^2^) and fed every third day until day 15 with EB2 media. All experiments began 30–54 days after the initiation of CM differentiation.

### Patterning

Microcontact printing and soft lithography were used to generate compliant substrates with extracellular matrix proteins in a defined pattern as previously described (Napiwocki et al., 2021a; Napiwocki et al., 2021b). Patterns were fabricated on a master Si wafer (FlowJEM, Toronto, ON, Canada) and used to produce reusable PDMS (Sylgard 184) stamps for microcontact printing. PDMS was poured onto the patterned wafer and cured overnight at 60°C then removed and cut into individual stamps. Stamps were coated with Matrigel (Corning) overnight before removing excess Matrigel and moisture with nitrogen. A polyvinyl alcohol (PVA) film was made by dissolving 0.5 g of PVA (Sigma Aldrich) in 10 ml of deionized water and dried overnight in a petri dish. Once dry, the PVA film with a thickness of 100 μm was removed from the petri dish and cut to sizes slightly larger than the stamps. The Matrigel coated stamps were brought into contact with the PVA film and allowed to transfer for 1 h in an incubator. To promote complete transfer, a glass slide and 50 g weight were placed on top of the stamp. After 1 hour, the PVA film was removed from the PDMS stamp and brought into conformal contact with the soft PDMS substrate. After 20 min the substrate was washed with PBS to dissolve the sacrificial PVA film, leaving behind the patterned proteins which were then seeded with cells. A small PVC tube was placed on top of the PDMS to control seeding of cells into the desired pattern region; seeding occurs on experiment day 0, defined as E0. The PVC tube was removed on the first day of the experiment, E1, and additional media added to bring the total volume to 2.5 ml per sample.

For experiment samples, PDMS substrates were made to a Young’s moduli of 10, 30, or 50 kPa by blending Sylgard 184 and Sylgard 527 (Dow Corning) (Palchesko et al., 2012). Sylgard 184 was made by mixing ten parts base to one part cure, and Sylgard 527 was made by mixing equal parts of components A and B. Both were mixed individually for 5 min with a glass stir rod and combined in a prescribed ratio to achieve the desired stiffness. The ratios of Sylgard 527 to Sylgard 184 were 52:1, 15:1, 10:1 for 10, 30 , and 50 kPa respectively. Once the components were mixed, PDMS was poured into 100 mm diameter petri dishes, allowed to sit under vacuum for 20 min to remove air bubbles, and cured overnight at 60°C. To overcome challenges associated with handling low modulus PDMS substrates, the PDMS was cured on top of a foundation layer of Sylgard 184 with a final thickness of 2 mm. After curing, the samples were cut to the desired size with a razor blade and UV sterilized prior to use.

### Microscopy

Brightfield videos of cells spontaneously contracting were acquired every other day starting on the third day, E3, until E15 using a Nikon Eclipse Ti microscope with a Plan Flour ×10 NA 0.3 objective and Nikon DS-QiMc camera with samples maintained at 37°C. At least two spontaneous contraction events were acquired for each sample at each timepoint with a video frame rate of 11.05 fps.

### Analysis

Mechanical analysis was done using previously developed open source software, with custom add-ons and modifications created for additional processing to desired outputs for this study (Napiwocki et al., 2021a; Napiwocki et al., 2021b; Notbohm et al., 2019). Using Fast Iterative Digital Image Correlation (FIDIC) (Bar-Kochba et al., 2015), displacements were computed relative to the first frame which contains cardiomyocytes in a relaxed state. The random high contrast pattern from the phase contrast images of the cells was sufficient to allow tracking of displacements. DIC was used to calculate displacements using the input parameters of a subset size of 48 × 48 pixels (31.2 × 31.2 μm) and subset spacing of 12 pixels. 2D displacements, U_*x*_ and U_*y*_, were computed for each frame relative to the initial frame. From the displacements, the normal strains in the *x* and *y* directions were calculated by taking the gradient of the displacement data. To compute the derivative, an optimal five element kernel in the *x* and *y* directions was generated and the derivatives of displacement found by taking a convolution (Farid and Simoncelli, 2004). To better understand strains that were likely to be along the axis of the vertical lanes and 15° bridges connecting the lanes due to the alignment of myofibrils within the cells, second principal strain, ε_2_ was calculated. The second principal strain (i.e., contractile strain) values were averaged for each frame of the video and the maximum magnitude of this averaged value over the course of the contraction event (i.e., maximum contractile strain) was used as a measure for comparison between sample conditions.

Contraction of CMs causes deformation across the entire substrate, therefore there were small strains in the regions of the sample where cells were not located. To eliminate strain values in unpopulated regions, a binary mask was used to eliminate data from areas not occupied by cells. Using previously developed boundary identification software, the boundaries within a full field image were found with edge detection techniques (Treloar and Simpson, 2013). Briefly, the edges of the cells in lanes were detected using a Sobel filter with a user-defined threshold. These edges were then dilated to form enclosed areas, and the inverse of these regions taken.

The spontaneous rate of contraction was computed for each sample. The average contractile strain for the entire video was plotted, and the peak average contractile strain was found. A line at 60% of the peak of the average contractile strain was plotted overlaid with the average contractile strain. For each contraction, the intersection points of the average contractile strain and 60% strain were found, and the midpoint between the two intersection points on each curve was identified. This was repeated for each contraction, for at least two contraction events per sample. The distance between the peaks was found and the distances were averaged for samples containing more than two contractions. The average distance in frames per beat was converted to seconds per beat by dividing by the frame acquisition rate (11.02 fps) and converting to beats per minute.

For statistical analysis of two groups an unpaired two sample *t*-test is used. For analysis with more than two conditions a one-way ANOVA test was performed followed by multiple comparison test to perform pairwise comparisons between pairs of data using Tukey’s honestly significant difference criterion. Statistical significance was defined as *p* < 0.05.

## Results

**FIGURE 1:**
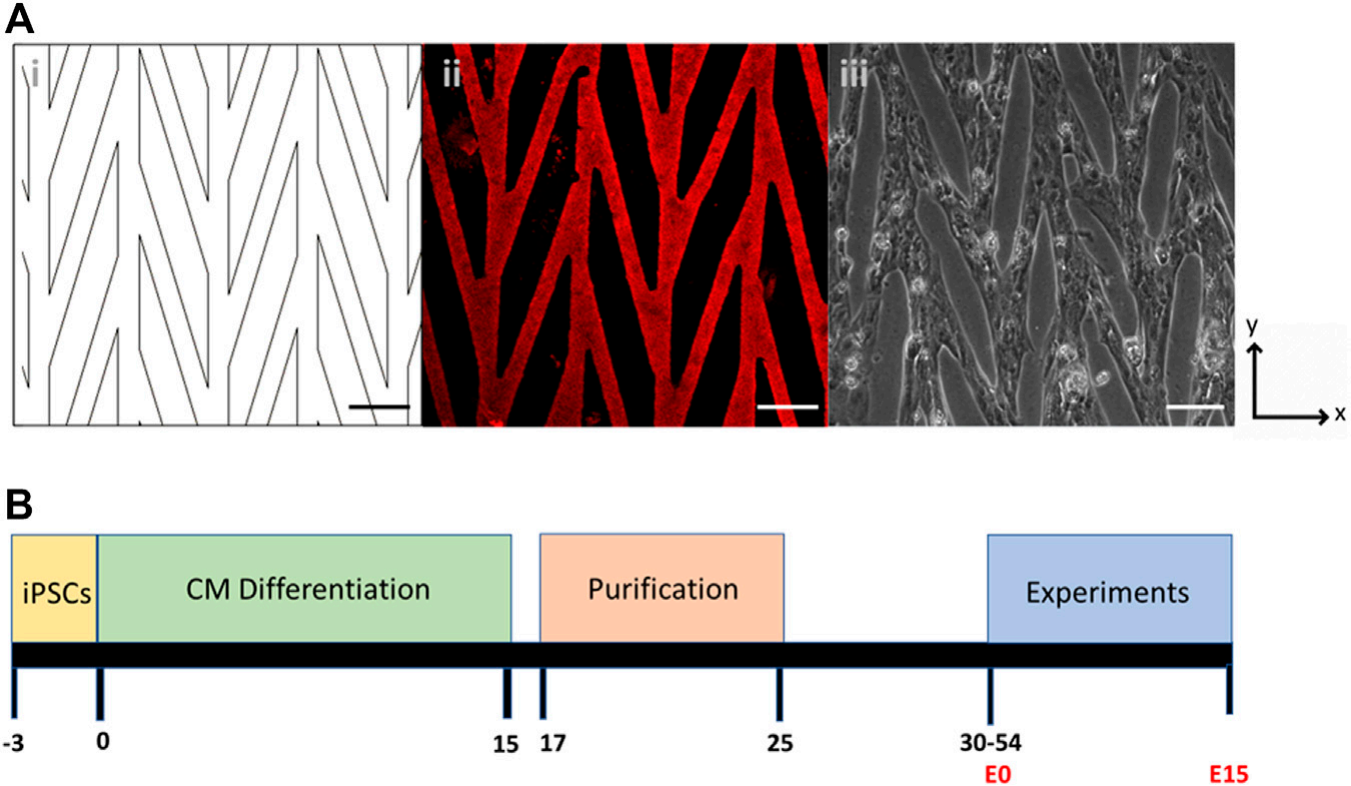
**(A)** Chevron pattern of 30 µm lanes connected at 15°: **(i)** CAD drawing of the pattern; **(ii)** Matrigel stained with laminin; **(iii)** Pattern seeded with control CPVT stem-cell derived cardiomyocytes. Scale bars are 100 µm. **(B)** Timeline of cell culture, differentiation, purification, and experiments. Day 0 denotes the start of CM differentiation and E0 denotes the start of experiments after cells are seeded onto a micropatterned substrate.

Two iPSC lines, one from the patient with CPVT (patient) and one from the patient’s mother (control) were differentiated into cardiomyocytes and purified before seeding. A brief patient history and pedigree are provided in Supplementary Results and Figure 1. Cells were seeded onto PDMS substrates coated with patterned Matrigel on experimental day 0 (E0) and cultured for 15 days (E15) with image and video acquisition throughout. Experiments were initiated between days 30–54 of the differentiation process. PDMS substrates with stiffness of 10, 30, and 50 kPa were used, which capture the range of stiffness from that of healthy to diseased myocardium; the experimental timeline is detailed in Figure 1B. For the patient cell line, five trials of 10 kPa, two trials of 30 kPa, and three trials of 50 kPa experiments were conducted. For the control cell line, three trials of 10 kPa, and two trials of both 30 and 50 kPa experiments were conducted. Within each trial there were multiple (2–7) samples for each cell line and multiple timepoints from day 3–15 (E3–E15). Samples where the pattern transfer was incomplete, or where lanes were merged such that cells behaved as a monolayer were excluded from analysis. The total number of biological replicates for the control line was 15, 8, and 5 for 10, 30, and 50 kPa respectively. Each replicate was imaged at multiple timepoints resulting in 98, 47, and 31 videos to be analyzed for 10, 30, and 50 kPa respectively. The total number of biological replicates for the patient line was 18, 9, and 16 resulting in 118, 61, and 89 total videos for 10, 30, and 50 kPa respectively.

A chevron pattern consisting of 30 µm lanes intersected at a 15° angle was used to provide connectivity across the pattern (Figure 1A). This pattern was chosen because it promotes sarcomere alignment through the syncytium, but also allows for large coordinated contractions across the sample (Notbohm et al., 2019). Bright field videos were captured for several contractions at timepoints between E3 thought E15 and analyzed with DIC (Supplementary Results, Supplementary Video S1). Displacements in the *x* and *y* directions were first computed using DIC. The contraction primarily occurs in the vertical direction, which is the orientation of the chevron pattern, and therefore the general alignment of the CMs. Although the major axis of the pattern aligns with the *y*-axis, the bridge regions that are 15° offset from vertical lanes contain cells whose contractions are not primarily in the *y* direction. To further quantify contraction and better capture the behavior of the cells in this orientation, the strain tensor was computed as described in the *Methods* section.

**FIGURE 2:**
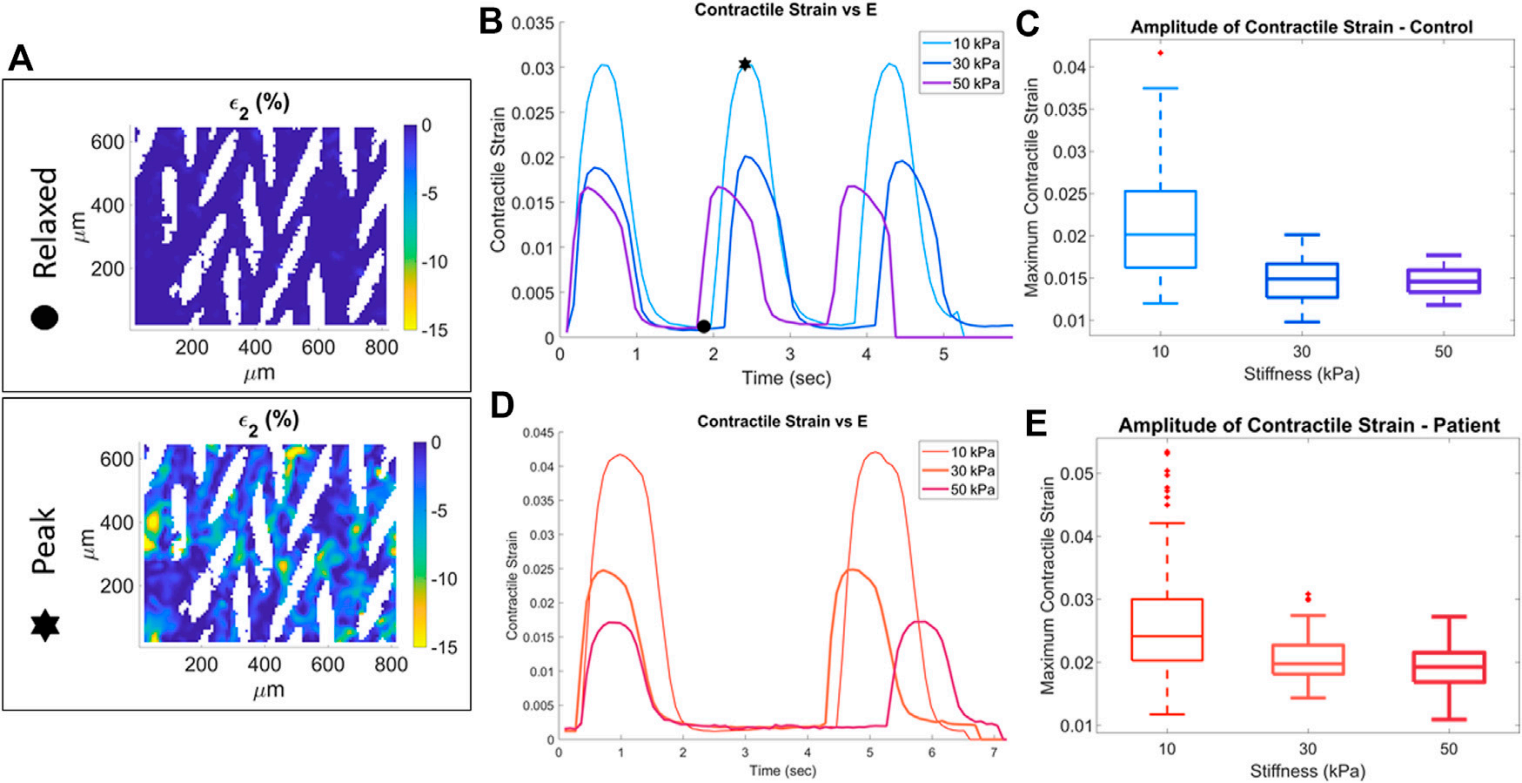
Quantification of contractile strain for control (blue) and patient (red) cell lines. Stars indicate relaxed state and peak contraction of a sample on a 10 kPa substrate. The full-field second principal strain for these two states is shown in the strain heatmaps **(A)**. Representative samples at each substrate stiffness for control **(B)** and patient **(D)** cell line. The maximum contractile strain for the three substrate stiffness conditions for the control **(C)** and patient **(E)** cell lines.

All cardiomyocytes contracting in the field of view were analyzed and the second principal strain ε_2_, i.e., contractile strain, was used as the quantitative measure of mechanical output (Supplementary Results, Supplementary Video S2). In the relaxed state, the contractile strain is ∼0% across the field of view whereas at peak contraction the contractile strain is as high as 15% in some locations (Figure 2A). To capture the behavior across the entire field of view the mean value is found for each frame of a video and used as the representative ε_2_ value. The frame average of the contractile strain is plotted over time (shown for convenience as magnitude |ε_2_|), for one representative sample each of 10 kPa, 30 kPa, and 50 kPa substrate modulus for the control cell line (Figure 2B). Indicators of relaxed and peak strain locations corresponding to the strain heatmaps in Figure 2A are shown on Figure 2B. In these representative samples the peak contractile strain decreases with increasing substrate stiffness. From the contractile strain vs. frame data, the maximum of the average values for each frame of each video is found, |ε_2_max_|. This results in one representative mean peak contractile strain value for each corresponding video for every sample; the entire dataset for all control cell line samples versus substrate stiffness is shown in Figure 2C. The peak contractile strain decreases as substrate stiffness increases. Similar analysis was done for the patient cell line; differences in contraction between 10, 30, and 50 kPa substrate modulus for representative samples are shown in Figure 2D. Similar to the control cell line, for the entire patient cell line dataset the peak contractile strain decreases as substrate stiffness increases (Figure 2E).

**FIGURE 3:**
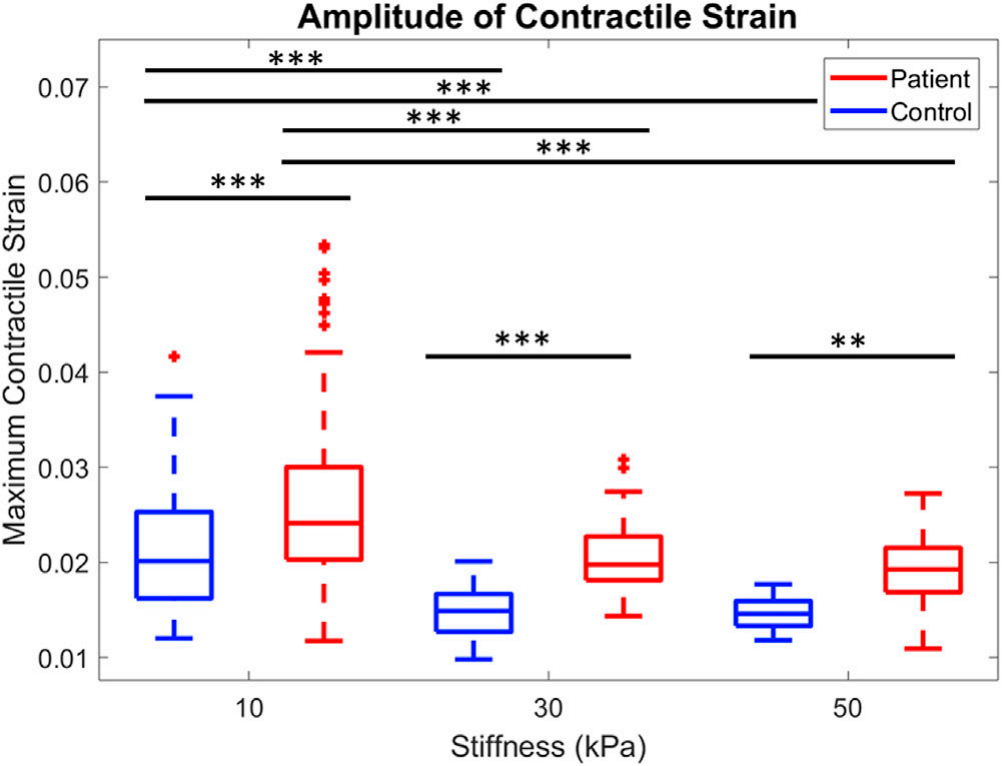
Comparison of peak contractile strain for control and patient across all three substrate stiffnesses are shown. **p < 0.001, ***p < 0.0001, one-way ANOVA with *post hoc* Tukey tests.

The maximum strain generated at each substrate stiffness was significantly higher in the patient line compared to control (Figure 3 **p < 0.001, ***p < 0.0001). Maximum contractile strain was 19.7%, 32.8%, and 24.3% higher in the patient line on 10, 30, and 50 kPa substrates, respectively.

**FIGURE 4:**
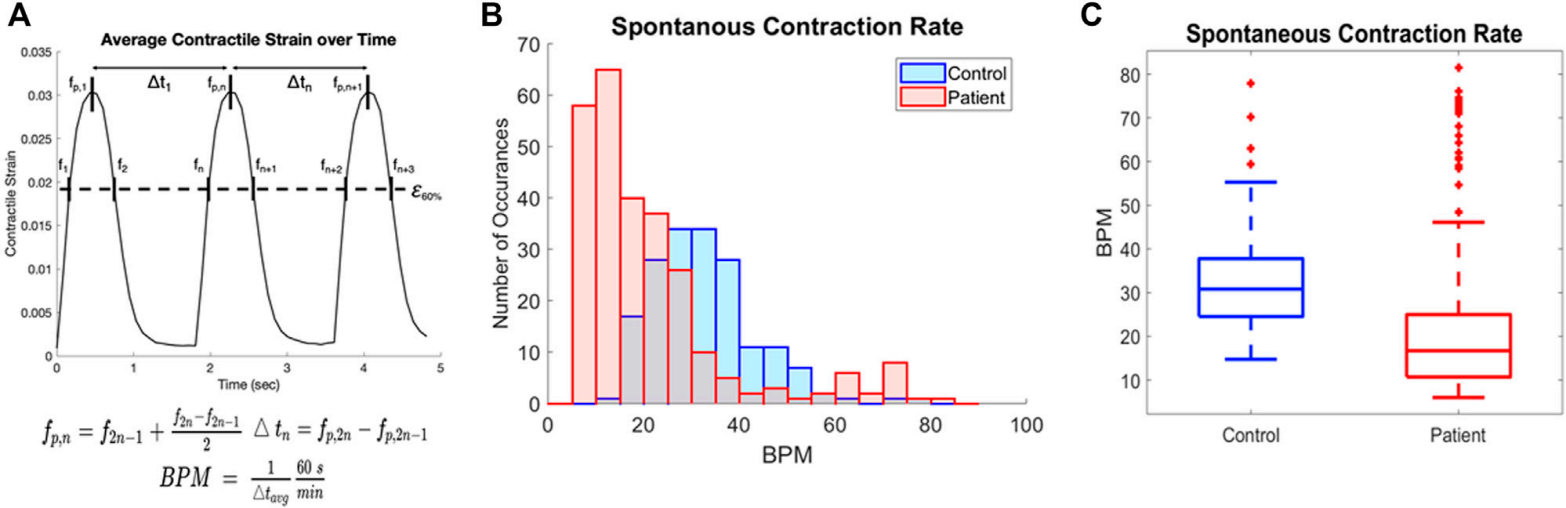
Quantification of contraction rate for patient and control cell lines **(A)** Contraction rate was calculated by the distance between mid-points of the intersection of 60% peak strain and average contractile strain plots and converted to beats per minute. **(B)** Overlaid histograms of the contraction rate for all samples of blue (control) and patient (red) cell lines. **(C)** Rate of spontaneous contraction across all samples is significantly slower in the patient line than the control *p* < 0.0001.

Spontaneous contraction rate can also be quantified from this data, and further analysis was done to quantify the rate of contraction for all samples. We determined the contraction rate by calculating the average time between contraction peaks and converting to beats per minute as shown in Figure 4A. The histogram for all patient samples generally shows a slower rate of contraction compared to the control samples (Figure 4B). The rate of contraction was significantly slower in the patient line than the control, with the mean contraction rate 45.7% lower in the patient line (Figure 4C, *p* < 0.0001). Notably, contraction rate and contraction strength are not correlated, thus the spontaneous contraction rate is not a confounding factor (Supplementary Figure S2).

## Discussion

Proper cardiomyocyte contraction, and thus contractile heart function, is a result of numerous complex interactions between cells and their environment. In understanding heart disease, it is useful to develop models in which environmental conditions can be controlled and modified to elucidate functional behavior. Mechanical analysis of CMs provides an opportunity to understand fundamental function of this cell type and quantify differences in conditions, such as in this work between a patient with CPVT and a non-affected gene-negative familial control. Prior results have explored behavior at the levels of individual cells (Kujala et al., 2012), unorganized monolayer (Preininger et al., 2016), and tissue constructs (Park et al., 2019), however the platform utilized here provides the opportunity to investigate cardiomyocytes in an organized syncytium. This culture platform bridges the gap between single-cell and tissue studies, by measuring contraction between multiple cells while including single-cell spatial resolution. Using microcontact printing to control placement of ECM proteins into defined patterns on compliant substrates has been previously shown to promote sarcomere alignment, increase cardiomyocyte elongation, recapitulate adult-like cardiomyocyte organization, and promote an anisotropic conduction velocity (Napiwocki et al., 2021a; Napiwocki et al., 2021b).

Numerous prior CPVT studies have focused on calcium handling, for example, refs. (Kushnir et al., 2010; Fatima et al., 2011; Novak et al., 2015; Ferrantini et al., 2016; Ahola et al., 2018). As a major calcium-handling channel involved in proper cardiac contraction, mutations of RyR2 often involve improper electrophysiological function and depressed contractility (Wehrens and Marks, 2003). *In vitro* studies have implicated RyR2 calcium mishandling with reduced peak of intracellular Ca^2+^ transient, as well as increased peak intracellular Ca^2+^ and slower diastolic decay of intracellular Ca^2+^ concentrations (Arai et al., 1993; Go et al., 1995), which could affect contractility. Overloading of cytosolic Ca^2+^ caused by SR leakage, as well as an inability to properly regulate reuptake, results in DADs (Viatchenko-Karpinski et al., 2004). β-adrenergic stimulation by the *in vitro* application of isoproterenol increased arrhythmogenesis through a mechanism potentially related to clinical CPVT observations (Fatima et al., 2011; Novak et al., 2015). Mechanical functionality of RyR2-linked CPVT has rarely been investigated, with analysis limited to peak tension in murine ventricular and atrial trabeculae (Ferrantini et al., 2016). In CPVT patients harboring RyR2 mutations, the heart is structurally normal and no consistent contractility phenotype is present. Most RyR2 mutations that trigger CPVT, including H2464D, are gain-of-function mutations implying greater Ca^2+^ release and/or increased intracellular [Ca^2+^], which may lead to increased contractility where there are no associated compensatory mechanisms.

Using an hiPSC line derived from a CPVT patient with the RyR2-H2464D mutation and a control line from the patient’s mother, mechanical analysis was performed using DIC on an engineered culture platform with substrate of Young’s modulus ranging from 10 to 50 kPa. Fast Iterative Digital Image Correlation (FIDIC) was used to evaluate displacements across collections of cells acting in coordination throughout contraction events. The full field displacement data was then used to calculate principal strains.

In these experiments, contractile strain was used as the key metric to compare differences between patient and control CPVT cell lines. In all three substrate stiffnesses tested, 10, 30, and 50 kPa, the patient CMs produced statistically significant higher contractile strains. Additionally, the patient line had a significantly slower intrinsic contraction rate than the control, which agrees with prior reports in literature using CPVT patient-specific stem cell derived CMs and also with clinical observations of bradycardia in CPVT patients (Postma et al., 2005). Further analysis showed that this difference in intrinsic rate was not correlated with maximum contractile strain or substrate stiffness.

Prior studies have shown that, in addition to normal patient ECG, contractile behavior in both animal and iPSC studies are normal at baseline conditions (Kushnir et al., 2010; Pérez-Riera et al., 2017; Ahola et al., 2018). This is consistent with our findings as no arrhythmogenic behavior was observed on this platform. We report on experiments conducted at baseline conditions, allowing for spontaneous contraction. Arrhythmogenic behavior in CPVT patients is generally observed with a heart rate above 120 BPM (Pérez-Riera et al., 2017); all samples in our experiments had a spontaneous contraction rate under 100 BPM. Treatment with isoproterenol has been demonstrated to result in arrhythmogenesis in both animal and hiPSC studies (Fatima et al., 2011; Bround et al., 2012; Novak et al., 2012). In our platform we anticipate this would overall increase contractility in both groups, but the percentage increase in peak contractile strain, beat rate, and presence of arrhythmogenic behavior may differ; future experiments would include these observations. Limitations of the study that would be important to explore in future studies are controlling beat rate through electrical pacing and repeating the analysis on iPSCs derived from other patients to understand if hypercontractility is characteristic of the disease. Additionally, differences in cardiomyocyte mechanical function may be sex-dependent, as the lines used in the study come from a male patient and the patient’s female mother. Dimorphisms in the cardiomyocyte transcriptome and calcium homeostasis indicate an altered mechanism of excitation-contraction coupling by biological sex (Parks and Howlett, 2013). In murine models, testosterone has been linked to differences in contractility, with male CMs having a more rapid contraction and relaxation than in females (Curl et al., 2009; Trexler et al., 2017). Greater left ventricular circumferential and longitudinal strains were observed in female organ-level human studies (Lawton et al., 2011; Augustine et al., 2013), but cellular measurements of strain in human cardiomyocytes are not well characterized. Future studies including a same-sex healthy familial donor will elucidate if hypercontractility is characteristic of the disease.

The finding of hypercontractility in the patient line under the conditions tested is a new result for CPVT. Differences in mechanical strain have not been previously reported, and hypercontractility is not a known characteristic of CPVT. However, this difference in mechanical function may represent an early disease phenotype. Stem cell derived cardiomyocytes are considered immature post differentiation, so the CMs in these experiments may be representative of an early disease timepoint rather than mature CMs which would better represent the stage at which the disease usually manifests. This implies there may be differences in development that should be further explored.

The results presented for a patient line containing a mutation causing CPVT compared to a control line derived from the patient’s mother show differences in contractile behavior. We observed the previously reported lower spontaneous rate of contraction in the patient line, demonstrating the utility of this approach in recapitulating features of cardiac disease (Novak et al., 2015; Ahola et al., 2018). While further analysis and testing of underlying mechanisms is required, the new results of increased contractile strain in patient CMs adds to the limited studies of mechanical function of CPVT CMs reported in the literature. This will inform future research in understanding CPVT disease presentation and progression.

## Data Availability

The raw data supporting the conclusion of this article will be made available by the authors, without undue reservation.
