# Supplementary material for: Identifying Features of Cardiac Disease Phenotypes Based on Mechanical Function in a Catecholaminergic Polymorphic Ventricular Tachycardia Model
Source: Front Bioeng Biotechnol. 2022 May 10;10:873531. doi: 10.3389/fbioe.2022.873531 (PMC9127198; doi:10.3389/fbioe.2022.873531)
Supplement: Supplementary file 2 [file DataSheet1.pdf]

Supplementary Material

1 Supplementary Data

Ethical Statement

Written informed consent was obtained from the patient in accordance with the last version of the Declaration of Helsinki and with approval by the University of Wisconsin Health Sciences Institutional Review Board also in accordance with the National Institutes of Health guidelines for human research.

Brief Clinical History

The patient presented at the age of 10 with exertional syncope. The patient's echocardiogram and ECG were normal, but epinephrine infusion induced bi-directional ventricular tachycardia (VT). Genotyping showed a RyR2 7390 C>G mutation, resulting in amino acid change H2464D. They were successfully treated with beta-blocker and follow up exercise tests showed minor ventricular ectopy only in recovery. Cessation of beta blocker or non-compliance correlated with recurrent events and he was exercise restricted since diagnosis. At age of 23 the patient suffered a cardiac arrest during sexual activity and beta-blocker was increased to metoprolol 50 BID plus flecainide 100 BID and an implantable cardioverter-defibrillator (ICD) was implanted. Subsequent strong emotional stress triggers have caused increased sustained and non-sustained ventricular fibrillation. The patient was switched to nadolol 120 daily plus flecainide 100 BID and has been event-free on this regimen. His mother is both asymptomatic and does not carry the H2464D mutation.

1.1 Supplementary Figures

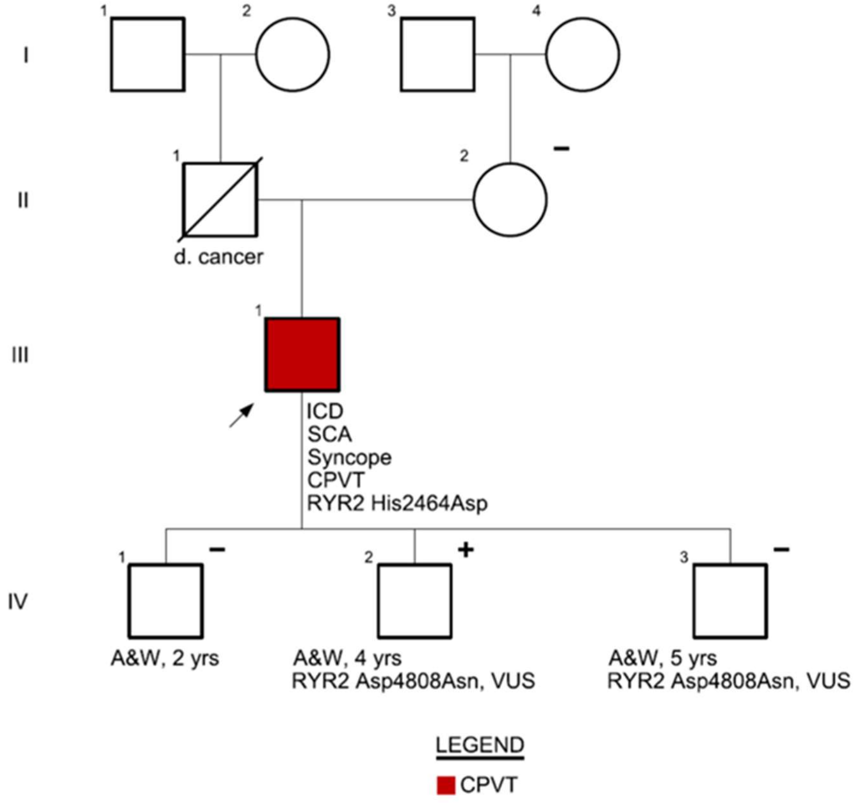

Supplementary Figure 1. Pedigree of the family; mother is genotype and phenotype negative and the patient harbors the mutation H2464D in RyR2 with symptoms of CPVT.

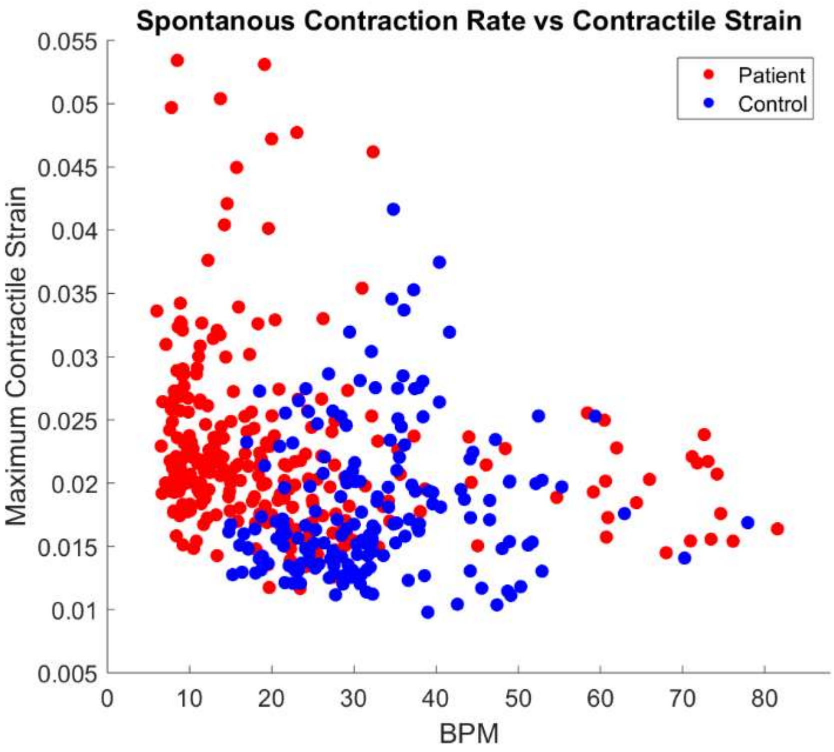

Supplementary Figure 2. Spontaneous contraction rate versus peak contractile strain for patient (red) and control (blue) cell lines. There is no correlation between spontaneous contraction rate and peak contractile strain for either cell line.

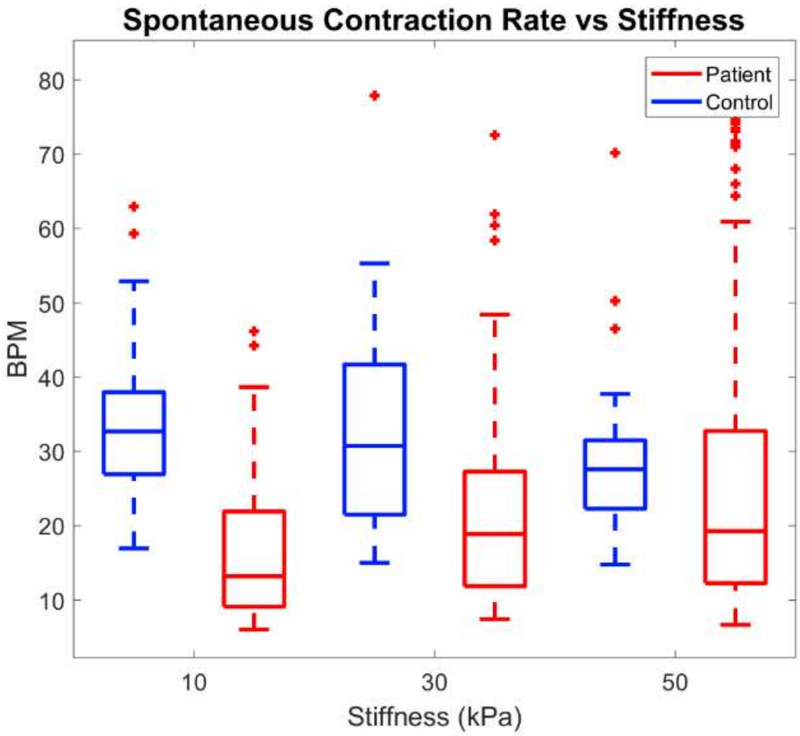

Supplementary Figure 3. Spontaneous contraction rate on 10 kPa, 30 kPa, and 50 kPa substrates for patient (red) and control (blue) cell lines. The spontaneous contraction rate is significantly lower in the patient line on all three substrate stiffnesses. There is no correlation between spontaneous contraction rate and substrate stiffness for either cell line.

Supplementary Video 1: Control iPSC-CMs contracting on 10 kPa PDMS. The entire field of view of a contraction event was used when calculating second principal strains for each condition.

Supplementary Video 2: Second principal strains for iPSC-CMs during a contraction event. Second principal strains are calculated for all video frames captured in a contraction event.
